# Structural and Functional Insights into Endoglin Ligand Recognition and Binding

**DOI:** 10.1371/journal.pone.0029948

**Published:** 2012-02-08

**Authors:** Aaron Alt, Laura Miguel-Romero, Jordi Donderis, Mikel Aristorena, Francisco J. Blanco, Adam Round, Vicente Rubio, Carmelo Bernabeu, Alberto Marina

**Affiliations:** 1 Instituto de Biomedicina de Valencia, Valencia, Spain; 2 Centro de Investigaciones Biologicas, Madrid, Spain; 3 Centro de Investigación Biomédica en Red de Enfermedades Raras, Instituto de Salud Carlos III, Valencia, Spain; 4 European Molecular Biology Laboratory, Grenoble, France; University of Oulu, Finland

## Abstract

Endoglin, a type I membrane glycoprotein expressed as a disulfide-linked homodimer on human vascular endothelial cells, is a component of the transforming growth factor (TGF)-β receptor complex and is implicated in a dominant vascular dysplasia known as hereditary hemorrhagic telangiectasia as well as in preeclampsia. It interacts with the type I TGF-β signaling receptor activin receptor-like kinase (ALK)1 and modulates cellular responses to Bone Morphogenetic Protein (BMP)-9 and BMP-10. Structurally, besides carrying a zona pellucida (ZP) domain, endoglin contains at its N-terminal extracellular region a domain of unknown function and without homology to any other known protein, therefore called the orphan domain (OD). In this study, we have determined the recognition and binding ability of full length ALK1, endoglin and constructs encompassing the OD to BMP-9 using combined methods, consisting of surface plasmon resonance and cellular assays. ALK1 and endoglin ectodomains bind, independently of their glycosylation state and without cooperativity, to different sites of BMP-9. The OD comprising residues 22 to 337 was identified among the present constructs as the minimal active endoglin domain needed for partner recognition. These studies also pinpointed to Cys350 as being responsible for the dimerization of endoglin. In contrast to the complete endoglin ectodomain, the OD is a monomer and its small angle X-ray scattering characterization revealed a compact conformation in solution into which a de novo model was fitted.

## Introduction

Endoglin, a TGF-β co-receptor expressed in endothelial cells, plays a key role in cardiovascular development, angiogenesis and vascular remodeling and homeostasis [1], [2], [3]. Mutations in the human ENDOGLIN gene (*ENG*) are responsible for Hereditary Hemorrhagic Telangiectasia (HHT) type 1, a disease characterized by frequent nose bleeds, telangiectases on skin and mucosa and arteriovenous malformations in lung, liver and brain [4], [5], [6]. Interestingly, ectodomain shedding of the membrane-bound receptor may occur under certain physiological conditions, and the plasma levels of this soluble endoglin form are highly increased and play a major pathogenic role in preeclampsia, a systemic syndrome of pregnancy which is associated with significant morbidity and mortality of both mothers and fetuses and which is characterized by pregnancy-associated hypertension and proteinuria [3]. Endoglin expression is markedly upregulated in proliferating endothelial cells, playing a crucial role in angiogenesis during development and in adult animals [1], [3], [7]. Indeed, endoglin knockout mice die *in utero* due to defects in the vascular system [8], [9], [10]. Cellular morphology, migration and adhesion [11] as well as cell responses to different members of the TGF-β family, including BMP-9 [12], [13] are modulated by endoglin. It has been postulated that endoglin's capacity to modulate TGF-β signaling is due to its ability to interact with the signaling type I receptors ALK5 and ALK1 and with the type II TGF- β receptor [5], [14], [15]. Several lines of experimental evidence support the notion that endoglin potentiates ALK1 signaling, including the fact that mutations in the gene coding for ALK1 (*ACVRL1*) give rise to a second form of HHT (HHT2) [5], [6]. Signaling triggered by the BMP-9-dependent ALK1/endoglin route mediates through the Smad1/5/8 pathway the expression of a vast number of genes, including the gene for the inhibitor of differentiation 1 (ID1), a negative transcriptional regulator which is associated with the development of malignant melanoma [2], [5], [14]. In addition, a crosstalk between the BMP-9/ALK1/endoglin route and that of TGF-β1/ALK5/Smad2,3 has also been reported in endothelial cells [2], [14], [16]. In this regard, ALK1 and endoglin are able to inhibit the TGF-β1/Smad3-mediated responses [1], [13], [15], [16], [17]. However, at variance with TGF-β1, BMP-9 has shown high specificity in ligand binding and signaling for endoglin and ALK1 receptors [18], [19], [20], [21]. Because of this specificity, BMP-9 has been postulated as the physiological ligand of the ALK1/endoglin pathway in HHT [12], [22].

Human endoglin is a 180 kDa homodimeric transmembrane protein that contains a 561- residue extracellular domain and a 47-residue serine/threonine-rich cytoplasmic region [23]. Structurally, endoglin belongs to the zona pellucida (ZP) family of extracellular proteins that share a ZP domain consisting of about 260 amino acids with eight conserved cysteine residues close to the transmembrane region [24], [25], [26]. This consensus ZP domain is located in the Lys362-Asp561 region of the endoglin ectodomain. Endoglin is highly glycosylated, in agreement with the reported potential O-linked glycosylation sites at Asn88, Asn102, Asn121, Asn134 and Asn307 [23]. The three-dimensional structure of the extracellular domain of endoglin at 25 Å resolution, determined by single-particle electron microscopy, showed that endoglin is arranged as a dome made of antiparallelly- oriented monomers enclosing a cavity at one end [25]. Each subunit comprises three well-defined regions, two of them corresponding to the ZP domains. The third region does not show any significant homology to other protein consensus motif/domain and thereby has been named the “orphan” domain (OD) [25]. Moreover, Small Angle X-ray Scattering (SAXS) experiments revealed an elongated conformation for soluble endoglin in solution, suggesting that endoglin might undergo conformational adaptations upon ligand binding [27]. A high resolution 3D structure remains to be determined. In this study, we have expressed recombinant versions of the extracellular full length domain of endoglin and of the OD. The ability of these constructs to recognize and bind BMP-9, both on their own account and in concert with ALK1, was examined by Surface Plasmon Resonance (SPR). Also, the recombinant OD of endoglin was structurally analyzed by SAXS experiments, revealing a compact conformation in solution into which a de novo model was fitted. While this manuscript was in preparation, a similar study by Castonguay and colleagues was published [28]. Therein the authors used immunoglobulin Fc domain tagged constructs, leading to slightly different results from ours, but reaching the same general conclusion, namely that the OD is responsible for ligand recognition and that binding of endoglin and ALK1 to their common ligand is unlikely to be cooperative.

## Results

### Soluble TGF-beta receptors are successfully expressed in HEK293 cells

**Figure 1:**
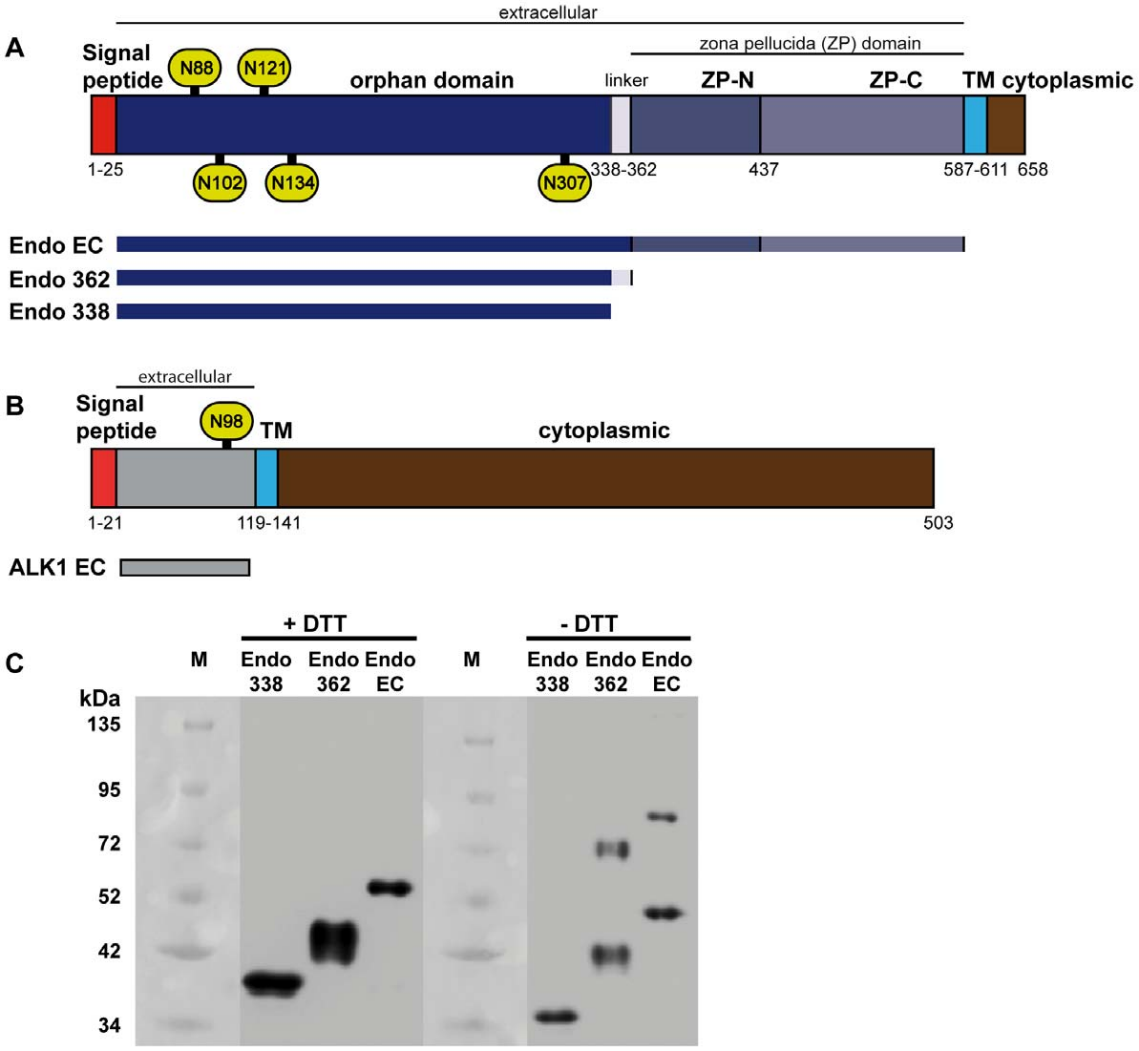
Schematic domain organization of human endoglin and ALK1 and western blots of endoglin domains. Bar diagram of (A) endoglin and (B) ALK1 with the domains indicated and highlighted in different styles. TM, transmembrane region, ZP, zona pellucida. The putative Asn glycosylation sites Asn88, Asn102, Asn121, Asn134 and Asn306 of endoglin and Asn98 of ALK1 are labelled within green ovals. The constructs used in this study and the domains encompassed by these are shown below the bar diagram of the respective full length protein. (C) Western blots of endoglin constructs. Endo_338_, Endo_362_ and LG-Endo_EC_ were analyzed by 10% SDS polyacrylamide electrophoresis gel followed by western blotting with an anti-His_6_ antibody. Samples reduced with dithithreitol (DTT) were incubated for 1 h with 10 mM of this reagent at 65°C. All samples (0.5 µg of protein) were then denaturated by boiling at 95°C for 5 minutes prior to charging onto the gel. The molecular weight markers (M) are indicated at the left of the samples. For both Endo_362_ and LG-Endo_EC_, dimeric species are visible, while Endo_338_ was only observed in monomeric form.

Previous results from our and other laboratories show that endoglin and ALK1 are not expressed well in *Escherichia coli* or in *Saccharomyces cerevisiae* [29], [30] where the produced protein is either not soluble or it aggregates and can only be purified in oligomeric forms which are unsuitable for structural and functional characterizations [31]. We therefore opted for expressing the ectodomains of these proteins in human cells, which should approach more the normal environment of these proteins and are capable of dealing with the necessary post-translational modifications to render correctly folded soluble proteins. Constructs for expressing the full length extracellular region of human endoglin (Endo_EC_, residues 22–587) and the truncated constructs Endo_338_ (residues 22–337) and Endo_362_ (residues 22–362) (Figure 1A) were cloned into the pOPING vector [32], which allows for secretion of the protein into the medium. The constructs were successfully overexpressed as soluble proteins in glycosylation-impaired HEK293S GnTI- suspension-grown cells, which produce glycoproteins with well defined sugars of the type Man_5_NAG_2_ [33]. Proteins produced in this way are thought to be more suitable for structural characterizations by protein crystallography or SAXS, since homogenous and well defined samples are a prerequisite for successful experiments with these two techniques. In order to assess the importance of glycosylation, Endo_EC_ was also overexpressed in HEK293 FreeStyle cells, which produce native full glycosylation. The proteins were purified from the media by immobilized metal ion adsorption chromatography followed by a gel filtration polishing step, resulting in a homogeneous protein, judged from Coomassie stained SDS gels. Typically we are able to purify 1.5–3 mg of protein per liter of cell culture.

Analogously to endoglin, a construct for ALK1 comprising the predicted complete ectodomain (AlK1_EC_ residues 21–118, Figure 1B) was cloned and purified for binding studies. ALK1_EC_ possesses a theoretical N-glycosylation site at Asn98. The approximate electrophoretic mass estimate for ALK1_EC_ produced in HEK293S GNT1- cells was 12 kDa, and upon treatment with EndoHf the mass was slightly reduced [34], due to enzymatic removal of the attached glycans (data not shown), indicating that ALK1 is indeed a glycoprotein.

In summary, our results demonstrate that the endoglin ectodomain OD can be produced successfully in a very homogenous soluble form when working with HEK293 FreeStyle cells (Figures S1A, S1B and S2), as can other well defined constructs of endoglin and ALK1 (data not shown).

### Identification of a disulfide bond dimerizing endoglin

A previous report identified the juxtamembrane Cys582 as being involved in the dimerization of endoglin via disulfide bonding [15]. However, the involvement of additional Cys residues in the dimerization process has been suggested [35]. Electrophoretical analysis under reducing and non-reducing conditions of Endo_EC_ and the OD constructs showed bands corresponding to dimeric and monomeric species for the full length endoglin ectodomain and for Endo_362_. In contrast, for Endo_338_ exclusively the monomeric form was observed in both redox states (Figure 1C). The native monomeric state of Endo_338_ was further confirmed by SAXS and analytical gel filtration (Figures S1B and S2). Previously the cysteine (or cysteines) responsible for the dimerization of endoglin was mapped to be located in the region between Phe282 and Ser431, harboring 6 possible cysteines (Cys330, Cys350, Cys363, Cys382, Cys394 and Cys412) that could be involved in the disulphide-mediated oligomerization process of the ectodomains [35]. The data presented herein therefore points to Cys350, being the only cysteine located in the region 338–361, as a residue clearly involved in the dimerization of endoglin (Figure S3).

### Evaluation of the contribution of glycosylation to ligand binding

**Figure 2:**
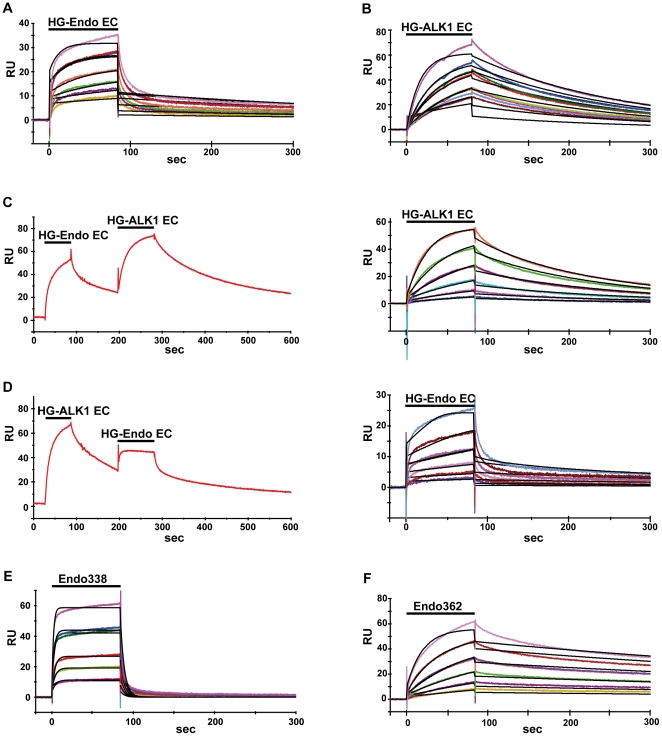
Functional interactions between endoglin, ALK1 and BMP-9. The interactions of (A) Endo_EC_, (B) ALK1_EC_, (E) Endo_338_ and (F) Endo_362_ with BMP-9 were investigated by SPR. While HG-Endo_EC_ and Endo_362_ dissociated slowly, Endo_338_ dissociated much faster, indicating a rigid body type of binding as opposed to an induced fit mechanism. For affinity measurements, the indicated recombinant proteins were injected at six concentrations ranging from 12.5 to 400 nM over BMP-9 (which was immobilized on a CM5 sensor chip by amine coupling) to generate sensorgrams (colored curves). When testing competition between HG-Endo_EC_ and HG-ALK1_EC_ (C and D) the chip was first pre-equilibrated with 750 mM of either HG-Endo_EC_ (C, left) or HG-ALK1_EC_ (D, left) before injecting the various concentration of the second ligand, showing the curve for the highest concentrations of the 2^nd^ ligand. Both HG-ALK1_EC_ (C, right) and HG-Endo_EC_ (D, right) yielded, after subtracting the background, similar results to those in runs in which no first ligand was preequilibrated before injecting the second ligand (D right vs. E; C right vs. B). This leads to the conclusion that endoglin and ALK1 bind independently to different sites on BMP-9. The kinetic parameters for the interaction were determined by global fitting (curves in black) of the 1∶1 Langmuir binding model to these data, providing values for the association (k_a_) and dissociation (k_d_) rate constants and the dissociation affinity constant (K_D_).

BMP-9 has been described as the ligand of ALK1 [18], [36] and endoglin [28]. In order to evaluate the importance of the glycosylation of these receptors for binding to BMP-9, we carried out surface plasmon resonance (SPR) binding assays with Endo_EC_ protein produced in both HEK293S GnTI-, which incorporates only short, well defined sugars rendering low glycosylated proteins (LG-Endo_EC_), and HEK293 FreeStyle, which produce mature, branched sugars and proteins with high glycosylation (HG-Endo_EC_) (Figure 2A). Sensor chip-immobilized BMP-9 bound LG-Endo_EC_ and HG-Endo_EC_ with apparent equilibrium dissociation constants (K_D_) of 2 nM and 10 nM respectively (Table 1, rows 1–2), indicating that recruitment of endoglin is mainly independent of the glycosylation state. The somewhat lower K_D_ value of the glycosylated endoglin form comes from both a lower avidity (k_a_∼0.5 times lower) and a faster dissociation (k_d_∼2.2-fold higher) (Table 1), suggesting that the sugar impairs to some extent the interaction of endoglin with BMP-9. This result also demonstrates that endoglin recognizes and binds BMP-9 in a highly efficient and selective manner on its own, at variance with other TGF-β family members that require the assistance of a type I TGF-β receptor [37], [38].

**Table 1:** Analysis of ligand binding to BMP-9 assessed by Surface Plamson Resonance.

	Analyte	Coinjection	Captured ligand	k_a_ (M^−1^ s^−1^)	k_d_ (s^−1^)	K_D_ (M)
1	HG-Endo_EC_	-	-	2.08E+5	2.07E−3	9.94E−9
2	LG-Endo_EC_	-	-	4.42E+5	8.91E−4	2.01E−9
3	HG-ALK1_EC_	-	-	1.21E+5	5.60E−3	4.64E−8
4	LG-ALK1_EC_	-	-	2.13E+5	4.03E−3	1.89E−8
5	HG-ALK1_EC_	-	HG-Endo_EC_	1.30E+5	7.72E−3	5.93E−8
6	HG-Endo_EC_	HG-ALK1_EC_	-	2.74E+5	1.53E−3	1.47E−8
7	LG-Endo_EC_	HG-ALK1_EC_	-	2.83E+5	9.25E−4	3.27E−9
8	HG-Endo_EC_	-	HG-ALK1_EC_	7.11E+5	1.09E−2	1.50E−8
9	LG-Endo_EC_	-	HG-ALK1_EC_	3.49E+5	9.13E−4	2.62E−9
10	Endo_338_	-	-	9.14E+5	2.54E−1	2.76E−7
11	Endo_338_	-	HG-ALK1_EC_	1.22E+6	1.92E−1	1.55E−7
12	Endo_362_	-	-	1.20E+5	1.08E−3	8.96E−9

Kinetic analysis of endoglin and ALK1 binding to BMP-9 was performed in triplicates on a Biacore T100 at 25°C as described in the experimental procedures. Data were globally fit to a 1∶1 binding model using the Biacore T100 evaluation software.

Next, the contribution of glycosylation in BMP-9 binding to ALK1 was assessed. ALK1_EC_ produced in HEK293S GNTI- (LG-ALK1_EC_) or HEK293 FreeStyle (HG-ALK1_EC_) cells was analyzed by SPR using BMP-9 immobilized on the surface of the sensor chip (Figure 2B). LG-ALK1_EC_ and HG-ALK1_EC_ bound to human BMP-9 with nearly identical values for k_a_ and k_d_ for both forms, resulting in K_D_ values of 46 nM and 19 nM respectively (Table 1, rows 3–4). Thus, the mature glycosylation of ALK1_EC_ seems not to affect the affinity for the substrate BMP-9.

### Assessing whether the binding of endoglin and ALK1 to BMP-9 is cooperative

Once having demonstrated that both receptors, endoglin and ALK1, recognize and bind individually with high affinity to BMP-9, we examined the possible cooperative nature of the interaction and binding process, using two different approaches. The first approach consisted in the capturing and saturation of the chip-immobilized BMP-9 with ALK1 prior to injection of endoglin at various concentrations (Figure 2C), while the second approach consisted of co-injecting endoglin with constant saturating concentrations of ALK1 onto the same chip. For both endoglin forms, LG-Endo_EC_ and HG-Endo_EC_, the K_D_ values obtained were mostly identical independently of the approach (Table 1, compare row 6 with 8 and 7 with 9). Both methods yielded K_D_ values that were closely similar to those estimated in the absence of ALK1 (Table 1, rows 1 and 2). We also carried out the converse experiment by saturating the BMP-9 immobilized chip with endoglin prior to injection of ALK1 at various concentrations (Figure 2D). Independently of the presence of endoglin, we obtained the same kinetic constants for ALK1 binding to BMP-9 (Table 1 rows 3 and 5). These results suggest that endoglin and ALK1 have different binding sites on their common ligand. Moreover, both receptors bind BMP-9 in an independent fashion, since no cooperative effect between endoglin and ALK1 was observed.

### The endoglin orphan domain is sufficient for recognition and binding of BMP-9

Sequence analysis of the endoglin ectodomain reveals a characteristic ZP domain that comprises the C-terminal portion (residues 363–561), remaining an N-terminal portion (residues 26–362) that does not show any significant homology to other protein consensus motifs and named, thereby, the “orphan” domain (Figure 1A) [25]. Since the ZP domain is a common motif present in a wide variety of proteins and serves to cluster and oligomerize surface receptors, including those involving TGF-β signaling [24], [26], [39], we hypothesized that the orphan domain could be the portion of endoglin that is involved in BMP-9 binding. To test this hypothesis, we produced two deletional endoglin constructs: Endo_362_, where the predicted ZP domain has been removed (Figure 1A), and Endo_338_, where a proline rich and predicted unordered region at the OD C-terminal part (Figure S3) was removed (Figure 1A). This small linker region (residues 338–362) connects the orphan and ZP domains and possibly allows for flexibility between these domains when binding to a ligand. It also carries Cys350 that our electrophoretical analysis identified as the possible mediator of endoglin dimerization via disulfide linkage. Both constructs were shown to bind BMP-9, although with quite different K_D_ values. Endo_362_ presents quite similar kinetic values to those calculated for the full endoglin ectodomain (Table 1, rows 2 and 12), with a K_D_ value of 9 nM. However, the K_D_ for Endo_338_ binding to BMP-9 was 270 nM, a value two orders of magnitude higher than the K_D_ for the binding of the complete ectodomain (Table 1, rows 2 and 10). This increase in the K_D_ is due to a faster dissociation of the Endo_338_ - BMP-9 complex, while keeping the same avidity for the ligand (Table 1, compare k_a_ and k_d_ values of rows 2 and 10). These results confirm that the recognition and binding of BMP-9 by endoglin fall on its orphan domain. We thus have delimited the minimum interacting region to the 22–338 fragment. Comparison of Endo_EC_ and Endo_338_ sensograms reveal that the former construct associates and dissociates more slowly to and from BMP-9 than Endo_338_ (Figures 2A and 2E). This suggests rigid body rather than induced fit binding in the case of the interactions between the orphan domain, represented by Endo_338_, and BMP-9. This effect is less striking for Endo_362_, which behaves like Endo_EC_ (Figure 2F). Thus the orphan domain is needed for recognition, while the ZP domains appear to stabilize the extracellular portion of endoglin, enhancing the stability of the complex, when formed [26], [39].

Taking into account the fast Endo_338_ - BMP-9 association and dissociation, we analyzed whether the presence of ALK1 could have any effect on this interaction. As in the case of the full length constructs, the presence of HG-ALK1_EC_ did not influence the binding nor the dissociation of Endo_338_ to or from BMP-9 (Table 1, rows 10–11), indicating that the presence of the ZP domain does not interfere with, nor contributes to BMP-9 binding.

### Competitive inhibition of signaling

**Figure 3:**
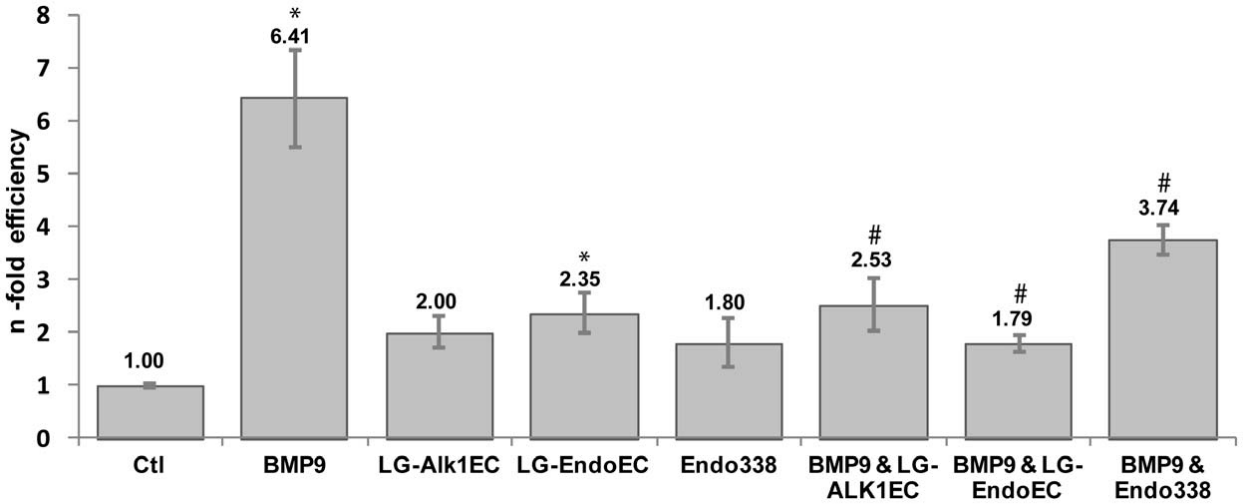
Functional analysis of recombinant endoglin and ALK1 proteins. ID1 expression in BMP-9 stimulated HMEC-1 cells after 36 hours in the presence or absence of LG-ALK1_EC_, Endoglin_338_ or LG-Endo_EC_. The ALK1 and endoglin ectodomains hijack BMP-9 and therefore signaling is diminished. Endo_338_ only partially inhibit signaling, due to a less stable complex formed between the orphan domain and the cytokine. (*) Statistically significant (p<0.01) difference compared to unstimulated control cells (Ctl). (#) Statistically significant (p<0.01) difference compared to ID1 expression of BMP-9 stimulated cells.

In order to evaluate the functionality of the proteins analyzed we checked the effect of ALK1 and endoglin on the response induced by BMP-9 in human microvascular endothelial cells (HMEC-1). It is known that BMP-9 activates, through the Smad1/5/8 pathway, the expression of *ID1* [40]. Therefore we measured by real time PCR the expression of this gene in cells treated with 75 ng/ml BMP-9 in the presence or absence of 10 µg/ml LG-ALK1_EC_, 10 µg/ml LG-Endo_EC_ and 10 µg/ml Endo_338_ as well as with only the receptors for 36 hours. As expected, stimulation of endothelial cells with 75 ng/ml BMP-9 induces a high (∼7 fold) increment of *ID1* expression. This effect is specific of BMP-9, since the supplementation with the recombinant receptors analyzed in the assay in absence of BMP-9 has an extremely weak (1–2 fold) effect on the expression of *ID1* (Figure 3). When co-incubating with BMP-9, both, LG-ALK1_EC_ and LG-Endo_EC_ diminish BMP-9 signaling to a large extent, bringing it close to the basal value observed when incubating the cells only with the ectodomains (Figure 3). This is likely due to the fact that LG-ALK1_EC_ and LG-Endo_EC_ are effectively sequestering BMP-9 and thus interfere with the downstream signaling. On the other hand, the addition of Endo_338_ only partially reduces the signaling. As learned from the SPR experiments, although Endo_338_ recognizes and binds to BMP-9 it also dissociates ∼100 times faster from its ligand (Table 1). This equilibrium favors the free state of BMP-9, increasing the pool of molecules that can stimulate *ID1* expression and, consequently, decreasing only about ∼50% the BMP-9 dependent signaling (Figure 3). These cell-based assays demonstrate that the constructs used in our experiments are functional, having the capacity to interact and to inhibit BMP-9-mediated signaling, and they confirm the orphan domain to be the BMP-9 recognition domain of endoglin.

### Low resolution structures of the orphan domain

**Figure 4:**
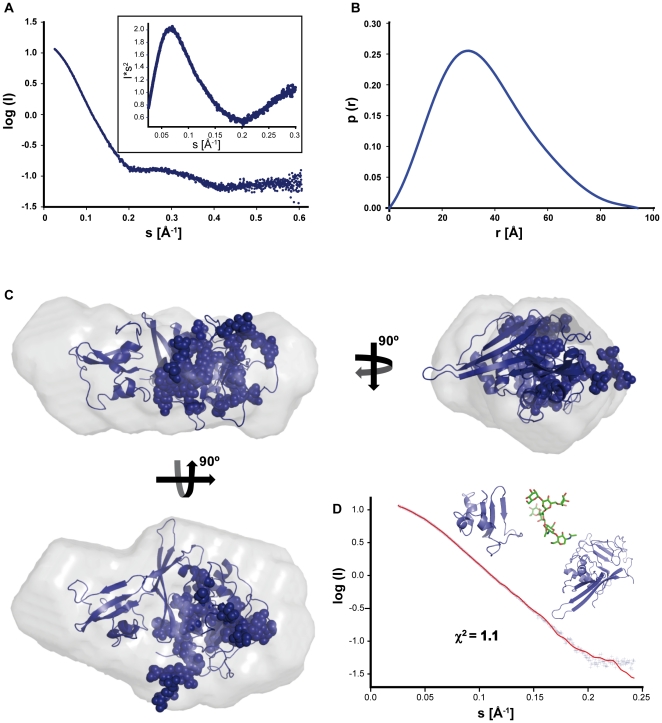
SAXS data analysis and rigid body modeling. (A) The experimental SAXS profile of recombinant Endo_338_, measured at the ID14-3 BioSAXS beamline. The Kratky plot (inserted), displays a gaussian behavior, which indicates the well folded nature of the construct. (B) The distance distribution function, p(r) obtained from GNOM with a D_max_ of 9.4 nm also suggests a compact structure. (C) The orphan domain model obtained from I-TASSER together with 5 Man_5_NAG_2_ glycans, restricted by their attachment sites was fitted by rigid body modeling with SASREF, yielding a fit of the model (red line) against the experimental Endo_338_ scattering profile (blue crosses) of χ^2^ = 1.1. The input models used, are depicted above the fit curve. (D) The resulting model is displayed as a cartoon in three orthogonal views, with the attached sugars depicted as balls, fitted into the molecular envelope from DAMMIF.

The solution structure of Endo_338_ was investigated by small angle X-ray scattering (SAXS). From the experimental scattering curve obtained from exposing a solution of Endo_338_ to X-rays (Figure 4A) the independent parameters for the radius of gyration (Rg) and the maximal dimensions (D_max_) of the orphan domain can be deduced. These parameters allow determining the oligomeric state, the overall shape of the molecule and to assess flexibility, which could arise from unordered regions. The Rg of 2.81 nm obtained from the Guinier plot (Figure S2A) and Dmax of 9.4 nm suggest that the protein has a compact conformation (Table 2 and Figure 4B). The relative molecular mass estimated from I(0) and the concentration of Endo_338_ yielded a molecular weight of 40 kDa through BSA calibration, confirming the monomeric state of Endo_338_ in solution (Figure S2B). Furthermore, the Kratky plot, a representation related to the flexibility of the molecule analyzed, presents a gaussian appearance (insert Figure 4A) that indicates a well folded nature of the construct. The overall envelope of Endo338 was calculated *ab initio* from its scattering profile using bead modeling programs (see Experimental section; the most typical shape is provided in Figure S2C), obtaining 20 final independent DAMMIF models that were aligned and compared with DAMAVER [41]. All models yielded recurrent features that correspond to an average envelope of Endo_338_ in solution (Figure 4C) with overall dimensions of about 95×55×30 Å and planar ellipsoid shape, which is consistent with a globular well folded domain. The validity of this averaged model was supported by the reproducibility of the independent reconstructions and the low normalized spatial discrepancy (NSD, having a value of 0.55) between them, and their good fitting to the experimental data (χ^2^ = 0.95). Furthermore, similar results were obtained with DAMMIN and GASBOR [41].

**Table 2:** Experimental and modelling SAXS parameters.

construct	Exp. I(0)^a^	Conc. (mg/ml)^b^	Estimated Mw (kDa)^c^	V_Porod_ (nm^3^)^d^	Mw (kDa)^e^	R_g_ f (gnom real space/gnom reciprocal/guinier)	D_max_ g (nm)	χ^2 (over)^	χ^2 (sasref)^
Endo_338_	13.6	3.7	40	67.8	39	2.82/2.81/2.81	9.4	0.952	1.1
BSA	12.4	4.85	66.0	147	66.3	3.11/3.1/3.1	10.7	ND	ND

a Values for I(0) have been extrapolated by the Guinier approximation from the experimental scattering profiles.

b Concentration of the protein used for the calculation of the estimated Mw.

c Relative molecular mass estimated from I(0) and the concentration of the protein through BSA calibration.

d The Porod volume was calculated using PRIMUS [41].

e Expected molecular mass predicted from the sequence and assuming full occupation of the glycosylation sites.

f Rg (Guinier), Rg (GNOM), radius of gyration given by the Guinier approximation, and calculated by the program GNOM, respectively, given in nm.

g Maximum dimension of the macromolecules. χ^2^ ^(over)^ Discrepancy between the SAXS profile and its fit by the overall shapes-models calculated by DAMMIF, and χ^2 (sasref)^ the average discrepancy of the best atomic models estimated with the program CRYSOL. ND, not determined.

With the purpose of clarifying whether the SAXS-deduced envelope could fit the OD fold, we tried to model the structure of this domain using existing programs for de novo model building, since comparative modelling could not be used because of the lack of OD homologues of known structure. We calculated de novo models using the online I-TASSER and Robetta servers [42], [43]. The best models (selected by their fit to the SAXS data evaluated with CRYSOL [41], see Figure S4) were used as starting models for further calculations. The outcome was an orphan domain fold comprised of 2 separate domains. These two domains, together with the five Man_5_NAG_2_ glycan chains (Figure 4D; modeled with the SWEET2 online server [44]) found on the proteins surface, were further fitted by rigid body movements using SASREF in the experimental SAXS envelope [41]. The best final model with a fit of χ^2^ = 1.1 is in extremely good accordance with the ab initio SAXS-deduced shape (Figure 4D), which consists of a ∼230-residue domain corresponding to the N-terminal part of the OD, which carries all the glycans, and which is followed by a smaller domain of ∼80 residues (Figure 4C).

## Discussion

Endoglin is classified as a type III TGF-β receptor, playing a crucial role in angiogenesis, preeclampsia and cancer. However, the complete functional and structural analysis of the endoglin domains remains to be elucidated. Recently, the ligands of ALK1, namely BMP-9 and BMP-10 were identified [18], [36], which therefore were also suggested to be the native ligands of endoglin, since both receptors were identified to be part of the same signaling complex. Moreover, mutations in both receptors result in HHT [4], [5], [6]. We therefore were interested in characterizing the binding of endoglin to its proposed ligand BMP-9 and to the complex ALK1 - BMP-9 in order to evaluate the putative cooperative nature of complex formation. Towards this aim, we expressed ALK1 and endoglin in a mammalian cell-based expression system, which also allowed us to fine-tune the glycosylation patterns of the proteins produced. Since endoglin is comprised of a ZP region and the so called “orphan domain” we designed, in addition to the full length domain, truncated constructs of the OD guided by secondary structure predictions. Endo_362_ terminates at the end of the OD, just before the predicted start of the ZP domain. Endo_338_, a slightly shorter construct, was designed to eliminate a predicted unordered linker region at the C-terminal end of the OD. Interestingly, the Endo_362_, as well as Endo_EC_, dimerized to some extent through a disulfide bond, while Endo_338_ was clearly a monomer. Detailed analysis mapped the cysteine involved in endoglin aggregation at the region between Phe282 and Ser431 which harbours 6 possible cysteines (Cys330, Cys350, Cys363, Cys382, Cys394 and Cys412) [35]. The results presented herein suggest that Cys350, located in the unordered linker region spanning residues 338 to 362 between the ZP domain and the OD, together with the reported juxtamembrane Cys582 [15], are involved in the dimerization of endoglin via disulfide linkages. However, further studies will be needed to confirm whether or not additional cysteines are involved in the endoglin dimerization process. Using SPR analysis we have demonstrated that the ectodomain of endoglin can directly bind BMP-9 in a highly efficient manner. This observation was corroborated by the *in vivo* functional assays, where the presence of either endoglin or ALK1 effectively inhibited BMP-9-induced downstream signaling. Furthermore, endoglin and ALK1 bind to different non-overlapping sites on their common ligand BMP-9 as could be demonstrated by SPR, where no change in binding kinetics was observed when either co-injecting Endo_EC_ with ALK1_EC_ or pre-equilibrating the BMP-9 bound CM5 sensor chip with ALK1_EC_. However, it should be remembered that these assays were undertaken with the receptors ectodomains. In the case of cell signaling with the full-length membrane-embedded receptors, it is conceivable that there may be differences with the *in vitro* assays in the binding of BMP-9 to the receptor, since the intracellular and transmembrane domains of endoglin and ALK1 might interact with each other and promote somewhat different conformations and modes of binding. In this regard, specific pairing interactions among BMP-9, ALK1 and endoglin within the cell have been suggested based on co-immunoprecipitation and cross-linking experiments [14], [19]. This in turn, also opens the possibility of cooperativity in the binding to BMP-9 within the cellular context, in agreement with recent *in vitro* data [36]. However, at variance with a recent report [28], our experiments do not support the existence of a synergic cooperation in the binding of these proteins *in vitro*.

We also were interested in studying the contribution of the sugars to recognition and binding of the receptors to their ligand, BMP-9. To accomplish this aim, we have set up a protocol to produce high amounts of well-folded and functional full-length ectodomains of both receptors in two cell lines that yield mature and minimal glycoforms. The disposition of high and low-glycosylated forms of both receptors allowed us to demonstrate that this post-transcriptional modification has little contribution to the recognition and binding of BMP-9. In agreement with this *in vitro* observation, glycosylated and PNGase F deglycosylated endoglin ectodomain produced in CHO cells have comparable effects in reported assays carried out in U937 cells [27]. Based on these data, we favor the hypothesis that although glycosylation is related to correct folding and the stability of endoglin and ALK1 at its extracellular localization [45], [46], it is not involved in BMP-9 binding to the mature receptor proteins.

We have dissected endoglin in order to identify which domain is involved in ligand binding and have found that the implicated region lies between residues 22 and 338, which encompasses the orphan domain. Endo_338_ possesses nearly identical recognition abilities as the full length protein, judged from the k_a_ value for this construct. However, the Endo_338_ – BMP-9 complex is not very stable as can be deduced from the high k_d_ in its monomeric form. The slightly longer Endo_362_ construct can be viewed as a dimeric OD. This construct was bound and remained in complex with BMP-9 in a similar fashion to the full length extracellular domain of endoglin. Therefore the ZP domain appears not to be involved in the binding process and may have a mere structural role at the cellular surface.

The report by Castonguay et al. [28], published while this manuscript was in preparation, presents important discrepancies with our work which might be attributed to the fact that the proteins in that study were expressed as chimeras with the immunoglobulin Fc domain. This type of chimeras usually yield soluble and stable proteins [47], which allowed them to carry out an exhaustive and deep analyses, however the presence of the Fc domain can introduce in some cases structural restrictions and rigidity as well as mediate unspecific interactions. Specifically to avoid similar problems, our studies were carried out with endoglin and ALK1 proteins that do not introduce extra amino acids, allowing us to clearly identify Endo_338_ as the domain that is active in BMP-9 binding. The SPR studies by Castonguay, used to detect endoglin interactions with its ligands and co-receptor, yielded conclusions that are coincident with those of our study on BMP-9 recognition by the endoglin orphan domain and on the independence of the binding sites for BMP-9 of endoglin and ALK1. However, Castonguay and collaborators stated that their constructs 26–329 and 26–332 showed no binding activity to BMP-9 by SPR, and in turn identified the region 26–359 to be the smallest of their constructs being active in binding. This activity was attributed to either a potential α-helix in the 352–358 region or to the need of a flexible linker between the OD and the Fc domain to allow BMP-9 binding. Since we find that Endo_338_ binds to BMP-9, the second possibility appears the most likely one for the OD-Fc fusion chimera, raising the question on whether Fc could influence other aspects of BMP-9 binding. Moreover, Castonguay and collaborators found that the OD (26–378 and 26–359 in their study) has higher affinity for BMP-9 than the full-length ectodomain [28] in contrast to our observations that this domain has similar (Endo_362_) or lower (Endo_338_) affinity than the full-length ligand (Table 1, rows 12 and 10). Again, the presence of the Fc domain, which is known to dimerize [47], could be affecting the kinetic parameters. Indeed, the K_D_ values calculated by these authors are three orders of magnitude lower than in our studies (Table 1), which might be related to the presence of the Fc domain. The alternative possibility that this difference reflects poor folding of the proteins used in our assays is contradicted by our SAXS results which reveal for Endo_338_ a molecular envelop that corresponds to a compact, well-folded monomer and suggested that Endo_338_ does not have any overhanging flexible and unordered ends, confirming the quality of the proteins used in our binding assays. Indeed, the functionality of the proteins used in the present studies was confirmed by cell-based assays in which both endoglin and ALK1 were found to inhibit BMP-9 induced signaling, as expected if they form stable non-functional complexes with BMP-9, preventing this ligand from binding to its membrane-bound receptors and the subsequent triggering of downstream signals. This *in vivo* cell assay also corroborated the existence of an interaction of the orphan domain with BMP-9, although these assays also provided evidence that the interaction was weaker than for the full-length ectodomain, since the signaling was not completely abolished by the orphan domain. One remaining question to be clarified is, if ALK1 and endoglin do bind to different sites on BMP-9, why then is signaling reduced when only one of the 2 proteins is added? We hypothesize that the productive signaling complex should involve the correct spatial location of both membrane-bound ALK1 and endoglin co-receptors. Therefore, when BMP-9 binds to either one of the 2 soluble ectodomains the cellular bound analogous domain cannot bind anymore to its site, and thus the membrane complex cannot be formed, which in turn inhibits downstream signaling. Further specific experiments, falling out of the scope of the present work will be required to validate this hypothesis.

## Materials and Methods

### Reagents

Materials for chromatography were from Fisher Scientific. Reagents for cell cultures were from Gibco and Lonza. Chemical reagents and primers were purchased from Sigma-Aldrich, EndoH_f_ from New England Biolabs and In-Fusion enzyme from Clontech. CM5 sensor chips, the amine coupling kit, ethanolamine and the P20 surfactant were from GE Healthcare. RNeasy kit was from Qiagen. iScript cDNA Synthesis kit and iQ SyBR-Green Supermix were from BioRad. Carrier free BMP-9 was from R&D Systems and FreeStyle HEK293F cells from Invitrogen.

### cDNA cloning

The full length endoglin ectodomain (Endo_EC_, residues 22–587), its OD (Endo_338_, residues 22–337 and Endo_362_, residues 22–361) and the ALK1 ectodomain (ALK1_EC_, residues 21–118) were PCR amplified using human endoglin and ALK1 cDNAs, as templates, and specific primers (see Sup. Table 1). Amplified fragments were purified and inserted into the pOPING vector for expression in mammalian cells using the In-fusion cloning system and selected clones were verified by DNA sequencing. In this expression vector, the constructs are fused to the μ-phosphatase secretion leader sequence and a C-terminal 6xHis tag. Cloning procedures and the over-expression of the plasmids for transient expression were carried out with *E. coli* DH5α.

### Protein expression and purification

Endoglin was expressed in glycosylation-impaired HEK293S GnT1- suspension adapted cells which produce homogeneous and lower molecular weight sugar chains (Man_5_NAG_2_) [33]. Suspension-grown HEK293S GNT1- cells in FreeStyle medium supplemented with 1% foetal calf serum (FCS) were cultured at 37°C under 5% CO_2_ atmosphere and shaking at 130 rpm on a Multitron-2 incubator shaker (Infors AG, Bottmingen, CH). Upon reaching a density of 1.5–2×10^6^ viable cells/ml, cells were transfected with 1 mg DNA per liter of culture using polyethylenimine (PEI) as the transfection reagent at a DNA∶PEI ratio of 3.5∶1. Four days after transfection, the culture supernatant containing the secreted protein was harvested by centrifugation at 5000× *g* for removal of cells and debris. Subsequent steps were at 4°C. An equal volume of PBS (pH 7.4) containing 2 mM reduced glutathione and 20 mM oxidized glutathione was added and the protein was loaded onto a 5-ml HisTrap column using a peristaltic pump in a closed circuit overnight. Thereafter, the column was washed with buffer A (100 mM Tris-HCl pH 8, 150 mM NaCl) supplemented with 20 mM imidazole and endoglin was eluted with 350 mM imidazole. Subsequent size exclusion chromatography was carried out in buffer A using a HiLoad 16/60 Superdex 200 column (GE Healthcare). The pure fractions (shown by SDS/PAGE), were pooled and concentrated by centrifugation in an Amicon concentrator to ∼20 mg/ml and flash frozen in liquid nitrogen. Analytic size exclusion chromatography was performed using a Superdex 200 10/300 GL size exclusion column (GE Healthcare), calibrated with standards (Sigma-Aldrich) using buffer A as the running buffer. ALK1 was expressed and purified in an analogous manner to endoglin, except that after the centrifugation step the culture supernatant was not diluted in PBS before application to the HisTrap column. Thereafter, the gel purification polishing step was performed using a HiLoad 16/60 Superdex 75 column (GE Healthcare). Both endoglin and ALK1 were also expressed in FreeStyle HEK293F cells. These cells produce mature glycans and the resulting protein was then purified in the same manner as the protein produced in HEK293S GnT1- cells [34].

### Cell culture and functional assays of recombinant proteins

Human microvascular endothelial cells HMEC-1 were cultured on gelatin pre-coated plates as monolayers in MCDB 131 medium supplemented with 10% FCS 2 mM L-glutamine, 1 ng/ml EGF, 1 µg/ml hydrocortisone and 100 U/ml penicillin/streptomycin (complete medium) in a NAPCO incubator at 37°C in a humidified atmosphere with 5% CO_2_. For the functional characterization of the recombinant proteins, inhibition experiments of the BMP-9-dependent *ID1* induction were carried out. HMEC-1 cells were grown in 6-well plates to 40% confluence and then treated with 75 ng/ml BMP-9 in the presence or absence of 10 µg/ml ALK1_EC_, 10 µg/ml Endo_338_ or 10 µg/ml LG-Endo_EC_, as indicated, for 36 hours in MCDB 131 basal medium supplemented with 2% FCS. For quantitative real time PCR analysis, total RNA was isolated from HMEC-1 cells using the RNeasy kit and was reverse-transcribed using iScript cDNA Synthesis kit. Then, 1 µl of cDNA was used as a template for real time PCR performed with specific *ID1* primers (Sup. Table 1) using the iQ SyBR-Green Supermix. Amplicons were detected using an iQ5 real time detection system (BioRad). Transcript levels were normalized to 18S levels (Sup. Table 1 for primers used). Triplicates of each experiment were performed. Statistical analysis was performed on the Data Desk package (version 4.0). Significance was estimated with analysis of variance (ANOVA) followed by Fischers LSD test for multiple comparisons.

### Surface plasmon resonance binding studies

All surface plasmon resonance experiments were carried out on a BiacoreT100 instrument (GE Healthcare). Bone morphogenetic protein 9 (BMP-9) was covalently immobilized via primary amino groups on a CM5 sensor chip surface. The amount of immobilized BMP-9 corresponded to 114, 253, 321 Response Units (RU) in channels 1, 2, and 3 respectively. Channel 4 on the same sensor chip, reserved for control runs, was treated in the same way as channels 1–3, but without BMP-9 immobilization. For all SPR measurements, the recombinant proteins were diluted in running buffer (10 mM HEPES pH 7.4, 150 mM NaCl, 3 mM EDTA and 0.05% detergent P20) and centrifuged immediately before the runs to minimize possible effects from nonspecific aggregation. The association was monitored by injecting different concentrations of the analytes into all 4 channels starting with the lowest analyte concentration. All experiments were conducted in triplicates at 25°C at a flow rate of 30 µL/minute. Samples were injected during 85 seconds to achieve steady-state binding and a subsequent 600 seconds were allowed for dissociation. Between injections surfaces were regenerated with 0.5 M NaOH at a flow rate of 30 µL/minute for 15 seconds. For capturing experiments, based on the Biacore T100 manual, the first analyte was injected at a concentration of 750 nM in running buffer for 60 seconds into all 4 channels at a flow rate of 10 µL/minute to allow for trapping by BMP-9. Thereafter, the second analytes were injected at different concentrations into all 4 channels starting with the lowest analyte concentration, as described above. Another approach consisted of coinjecting the various samples together with a constant concentration of the competing analyte. All curves were corrected for nonspecific binding by subtraction of control curves obtained from injection of the corresponding protein through the blank flow channel. Both, the affinity and dissociation constants were calculated from the plots of the steady-state binding as a function of protein concentration, using the Biacore T100 evaluation software (Biacore AB) and a 1∶1 binding kinetic model (Table 1).

### Structural modeling of endoglin

The online servers I-TASSER [42] and Robetta [43] were used to derive a model for the extracellular domain of endoglin, comprising of residues 22–337. The 3D models of saccharides were modeled with the SWEET2 online server [44]. Figures were prepared with PyMOL [48].

### Small angle X-ray scattering (SAXS) experiments

Synchrotron X-ray scattering data were collected at the ID14-3 BioSAXS beamline (ESRF, Grenoble) [49]. Endo_338_ was measured at several concentrations ranging from 0.6 to 3.7 mg/ml. SAXS data were recorded at 25°C using a Pilatus 1 M detector at a sample to detector distance of 2.43 m, covering the range of momentum transfer 0.05<s<0.5 Å^−1^ (s = 4πsin(θ)/λ where 2θ is the scattering angle and λ = 0.931 Å is the X-ray wavelength). To assess radiation damage, ten successive 10 sec exposures of protein solutions were compared.

Data processing steps were performed using standard procedures implemented in the software package PRIMUS [41]. The forward scattering I(0) and the radii of gyration Rg were evaluated using the Guinier approximation, assuming that at very small angles (s<1.3/R_g_) the intensity can be represented as I(s) = I(0)exp[−(sRg)^2^/3]. The maximum dimensions (D_max_) were computed using the indirect transform package GNOM [41], which also provides the distance distribution functions p(r) (Figure 4B). The molecular weight (MW) of the solute was evaluated by comparison of the forward scattering with that of a reference solution of bovine serum albumin. The excluded volume of the hydrated particle was computed using a standard equation (Table 2).

#### Ab Initio modeling of the overall shapes

The overall shapes of all assemblies were restored from the experimental data by three independent programs: DAMMIF (20 runs), DAMMIN (10 runs) and GASBOR (10 runs) [41] with no symmetry restriction. The scattering profiles were used up to s_max_ = 0.17 Å^−1^ for Endo_338_ (Figure S1D). The low-resolution models obtained from different runs were compared using the program DAMAVER to give an estimate of the reproducibility of the results inferred from the *ab initio* shape calculation [41]. The damstart model, an output from the DAMAVER runs, was used in DAMMIN as a starting model in order to obtain the final model (Figure S2C).

#### Rigid body refinement of the models

*De novo* models obtained from the I-TASSER and Robetta servers were compared to the experimental SAXS data using CRYSOL [41]. Theoretical SAXS curves were calculated from the individual models lacking the glycans and their similarities to the experimental SAXS curves were quantified using the χ^2^ value between the theoretical and experimental curves. The best models were elected to be further fitted by rigid body modeling. Final models were then refined together with the 5 Man_5_ NAG_2_ glycans, restricted by their attachment sites with SASREF (Figures 4C and D).

## Supporting Information

Figure S1 **SAXS analysis of Endo_338_ and the** ***ab initio*** **bead model.** (A) Guinier analysis of Endo_338_ SAXS data shows linearity in the Guinier region, indicating lack of aggregation in the sample. (B) Mass estimated from the Porod volume (67800 Å^3^) indicates that Endo_338_ has an estimated mass of 34–45 kDa, which is in good agreement with the calculated mass of 39 kDa for the fully glycosylated monomeric species. (C) The damstart file, resulting from 20 independent DAMMIF runs was used as a start model for the subsequent DAMMIN run. The resulting *ab initio* bead model is displayed in three orthogonal views. (D) The curve (red) generated with DAMMIN up to s_max_ = 0.17 Å^−1^ fits really well (χ^2^ = 0.952) the experimental data (blue).
(TIF)

Figure S2 **Analytic size exclusion chromatography.** The molecular weight of Endo_338_ in solution was estimated from the elution profile using a calibrated Superdex 200 10/300 GL size exclusion column (GE Healthcare) using buffer A (100 mM Tris-HCl pH 8, 150 mM NaCl) as the running buffer. Endo_338_ eluted at 15.93 ml, corresponding to ∼40 kDa mass (based on column calibration with appropriate standards). This is consistent with the theoretical molecular weight of a monomeric and fully glycosylated species of 39 kDa (open red diamond labeled 39 shown within the inserted calibration curve).
(TIF)

Figure S3 **Secondary structure prediction for the orphan domain C-terminal region.** Secondary structure prediction and disorder prediction generated by the Phyre server [50] yield a consensus for the region 352–358, highlighted in grey, to be most likely of an unstructured nature, although PSIPRED (http://bioinf.cs.ucl.ac.uk/psipred/) predicts a very short helix for this region. The residues where the constructs Endo_338_ and Endo_362_ were truncated are shown in red, and Cys350 possibly contributing to the disulfide-mediated dimerization is shown in purple.
(TIF)

Figure S4 **Validation of the Endo_338_** ***de novo*** **models.** Models for Endo_338_ comprising residues 22–337 (1A–5A) were calculated with the online servers I-TASSER [42] and Robetta [43]. Discrepancy between the theoretical scattering curves of the models (1B–5B, red curves) and the experimental SAXS profile (1B–5B, blue curves) were evaluated using the program CRYSOL. Model 3, which yielded the best χ^2^ fit, was used for further modeling.
(TIF)
