# Supplementary figures and images for: Structural and Functional Insights into Endoglin Ligand Recognition and Binding
Source: PLoS One. 2012 Feb 8;7(2):e29948. doi: 10.1371/journal.pone.0029948 (PMC3275592; doi:10.1371/journal.pone.0029948)

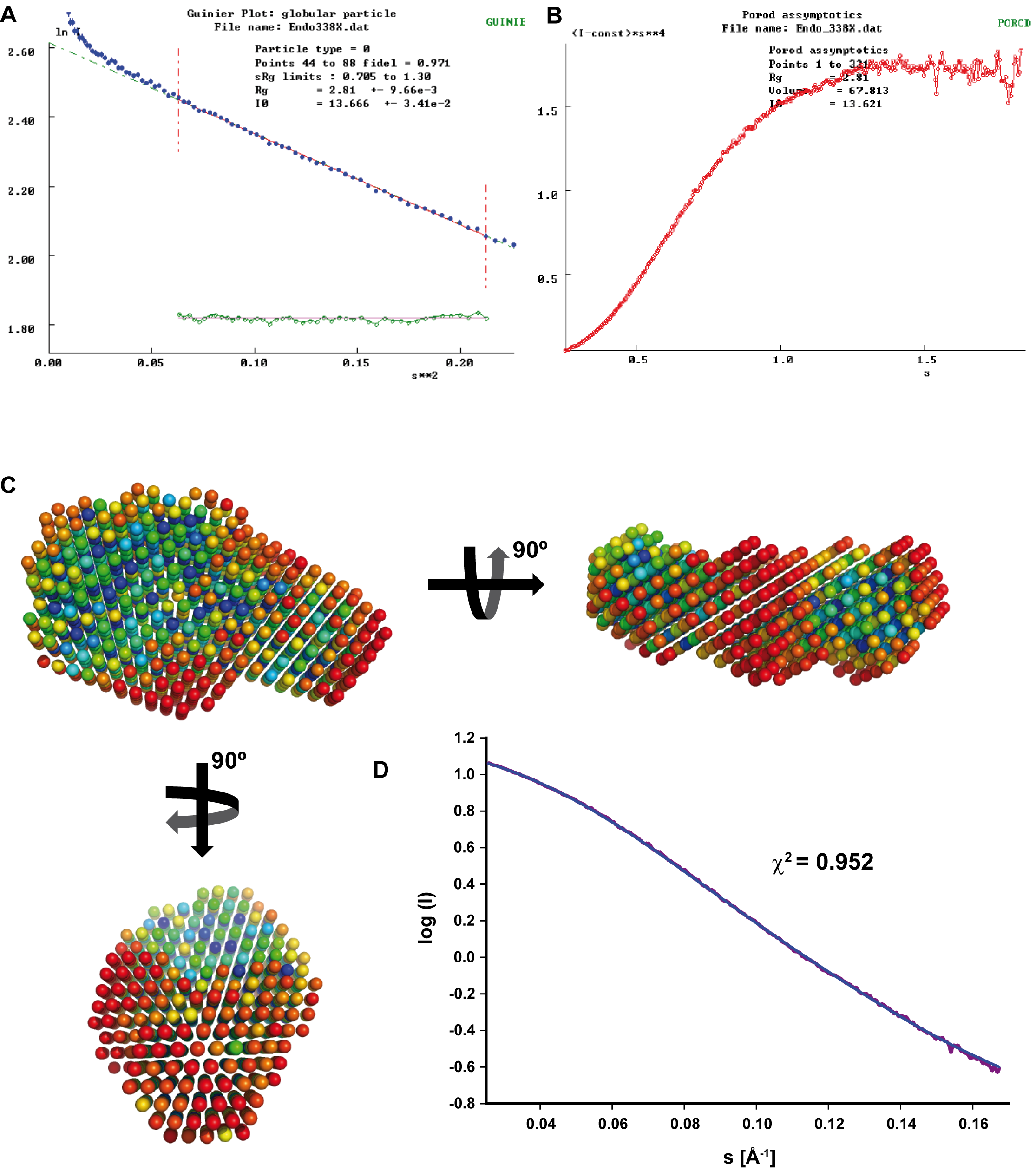

Supplement: Figure S1 — SAXS analysis of Endo338 and the ab initio bead model. (A) Guinier analysis of Endo338 SAXS data shows linearity in the Guinier region, indicating lack of aggregation in the sample. (B) Mass estimated from the Porod volume (67800 Å3) indicates that Endo338 has an estimated mass of 34–45 kDa, which is in good agreement with the calculated mass of 39 kDa for the fully glycosylated monomeric species. (C) The damstart file, resulting from 20 independent DAMMIF runs was used as a start model for the subsequent DAMMIN run. The resulting ab initio bead model is displayed in three orthogonal views. (D) The curve (red) generated with DAMMIN up to smax = 0.17 Å−1 fits really well (χ2 = 0.952) the experimental data (blue). (TIF) [file pone.0029948.s001.tif]

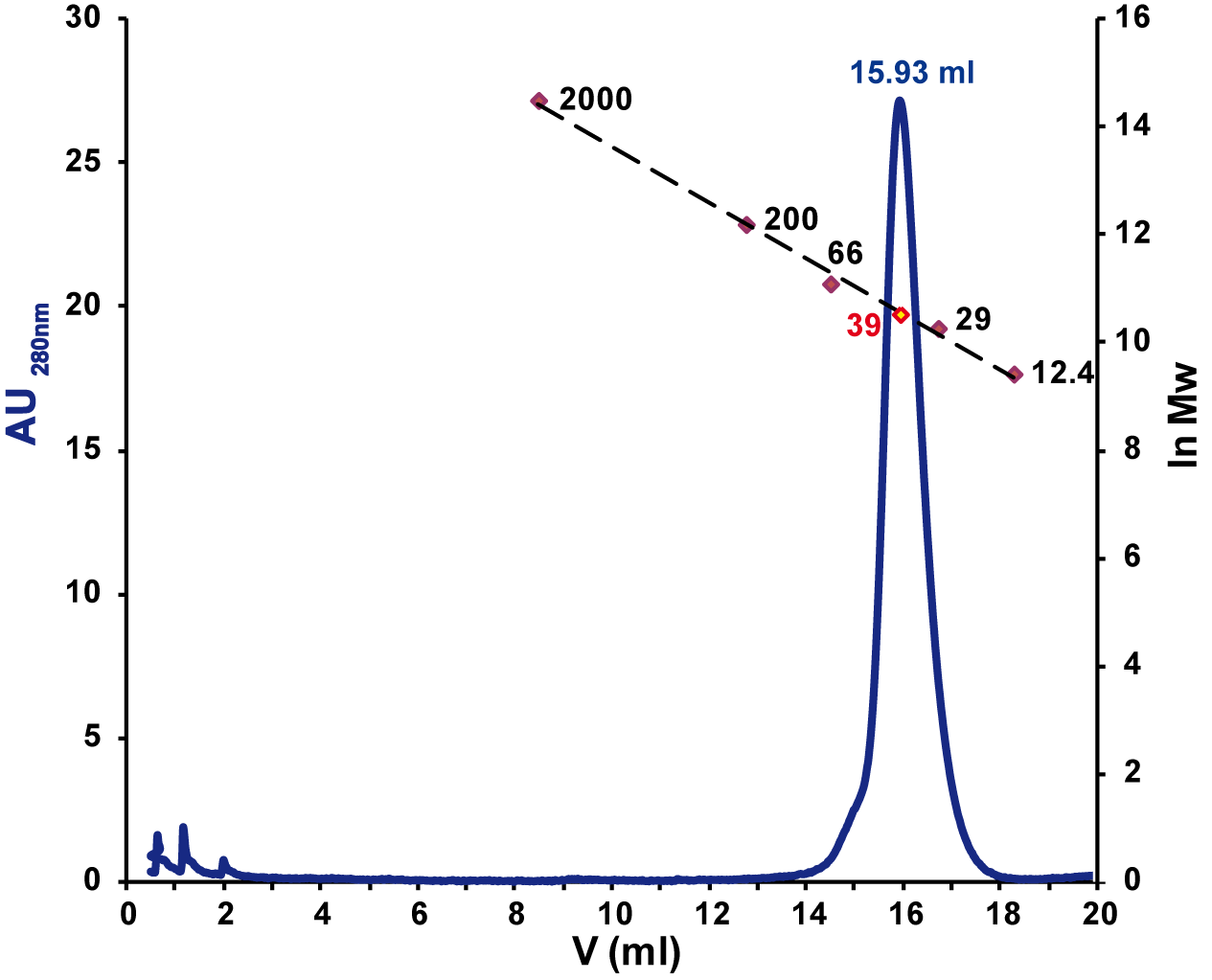

Supplement: Figure S2 — Analytic size exclusion chromatography. The molecular weight of Endo338 in solution was estimated from the elution profile using a calibrated Superdex 200 10/300 GL size exclusion column (GE Healthcare) using buffer A (100 mM Tris-HCl pH 8, 150 mM NaCl) as the running buffer. Endo338 eluted at 15.93 ml, corresponding to ∼40 kDa mass (based on column calibration with appropriate standards). This is consistent with the theoretical molecular weight of a monomeric and fully glycosylated species of 39 kDa (open red diamond labeled 39 shown within the inserted calibration curve). (TIF) [file pone.0029948.s002.tif]

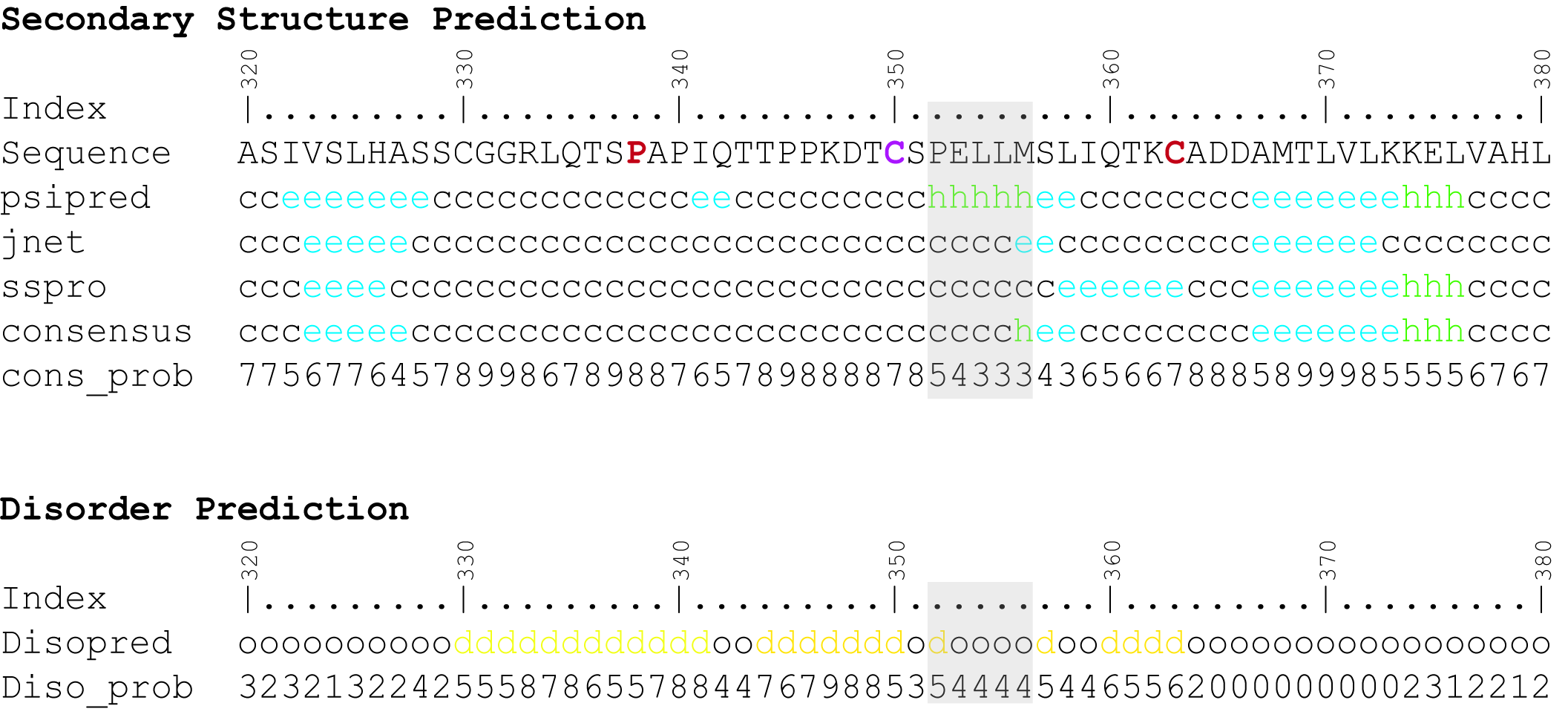

Supplement: Figure S3 — Secondary structure prediction for the orphan domain C-terminal region. Secondary structure prediction and disorder prediction generated by the Phyre server [50] yield a consensus for the region 352–358, highlighted in grey, to be most likely of an unstructured nature, although PSIPRED (http://bioinf.cs.ucl.ac.uk/psipred/) predicts a very short helix for this region. The residues where the constructs Endo338 and Endo362 were truncated are shown in red, and Cys350 possibly contributing to the disulfide-mediated dimerization is shown in purple. (TIF) [file pone.0029948.s003.tif]

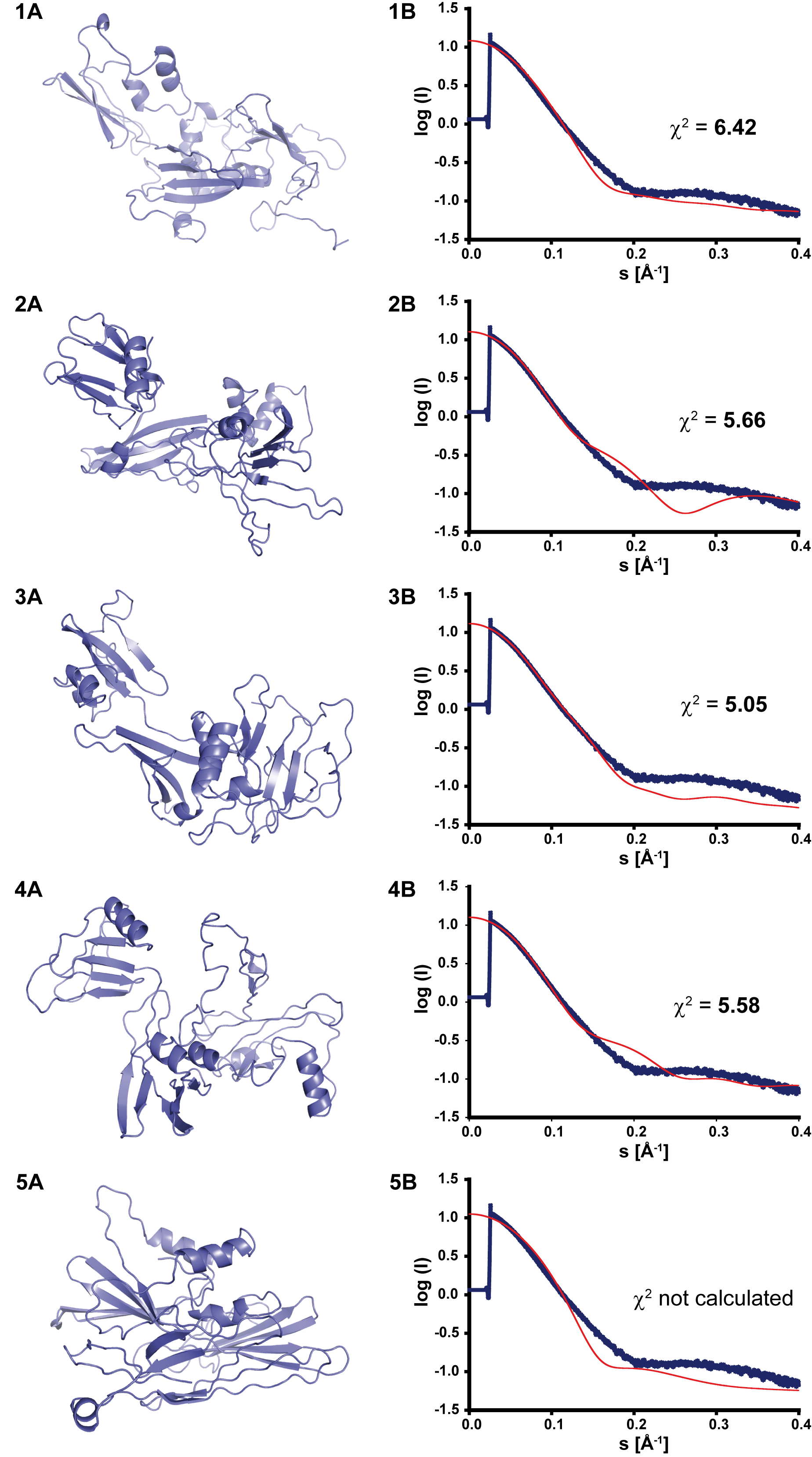

Supplement: Figure S4 — Validation of the Endo338 de novo models. Models for Endo338 comprising residues 22–337 (1A–5A) were calculated with the online servers I-TASSER [42] and Robetta [43]. Discrepancy between the theoretical scattering curves of the models (1B–5B, red curves) and the experimental SAXS profile (1B–5B, blue curves) were evaluated using the program CRYSOL. Model 3, which yielded the best χ2 fit, was used for further modeling. (TIF) [file pone.0029948.s004.tif]
